# Supplementary figures and images for: Diagnostic and Prognostic Potential of MiR-379/656 MicroRNA Cluster in Molecular Subtypes of Breast Cancer
Source: J Clin Med. 2021 Sep 9;10(18):4071. doi: 10.3390/jcm10184071 (PMC8467195; doi:10.3390/jcm10184071)

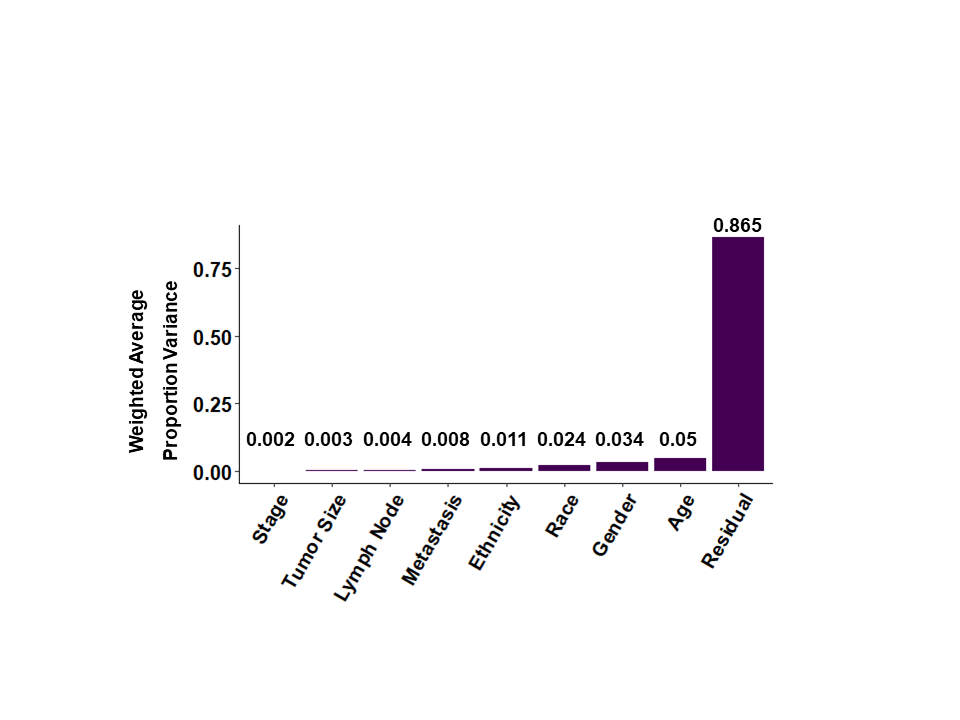

Supplement: Supplementary file 1 [file jcm-10-04071-s001.zip › jcm-1234077-supplementary/Supplementary Material Figure S1.PNG]

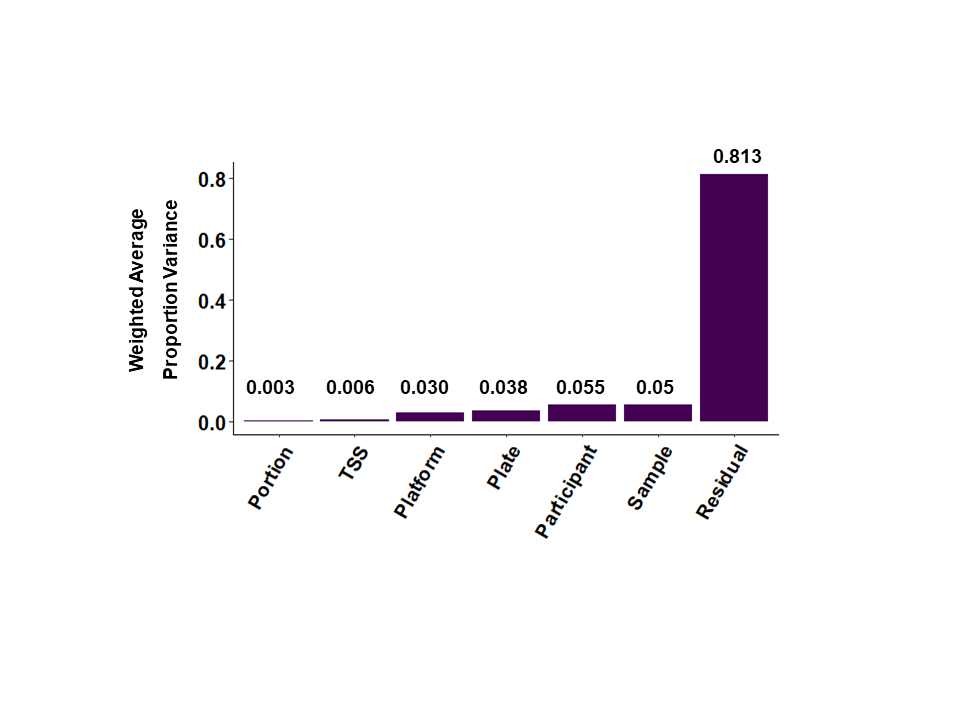

Supplement: Supplementary file 1 [file jcm-10-04071-s001.zip › jcm-1234077-supplementary/Supplementary Material Figure S2.PNG]

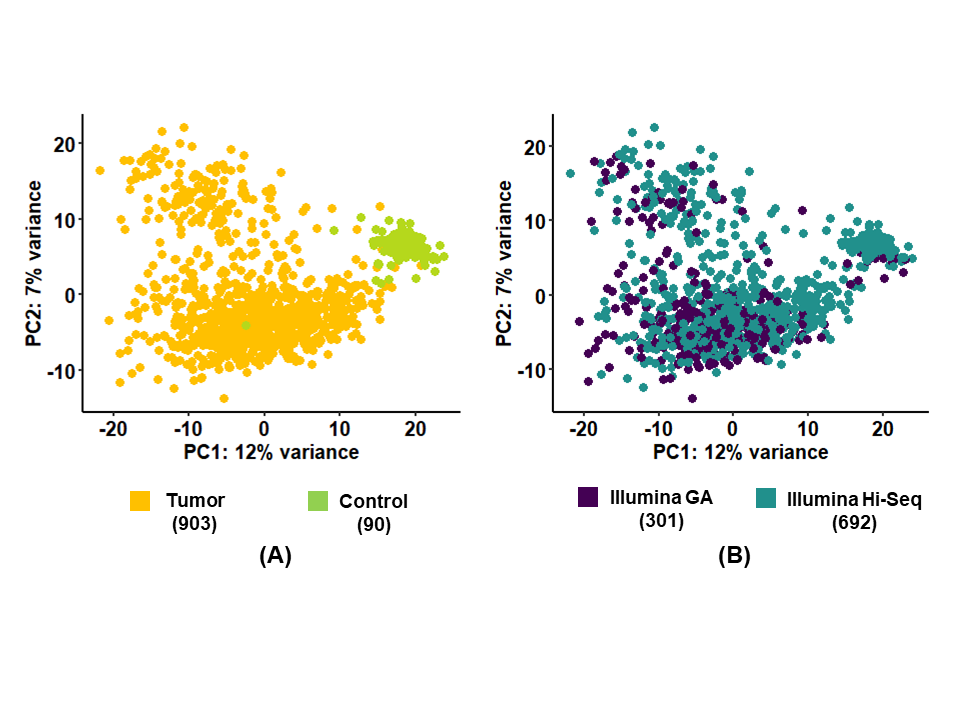

Supplement: Supplementary file 1 [file jcm-10-04071-s001.zip › jcm-1234077-supplementary/Supplementary Material Figure S3.PNG]

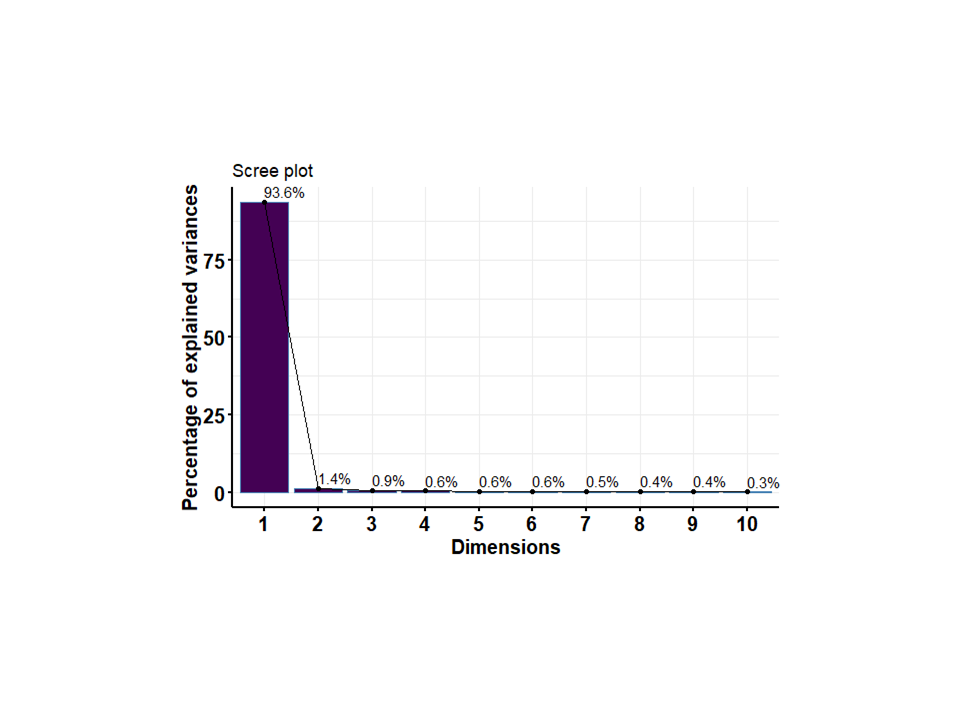

Supplement: Supplementary file 1 [file jcm-10-04071-s001.zip › jcm-1234077-supplementary/Supplementary Material Figure S4.PNG]

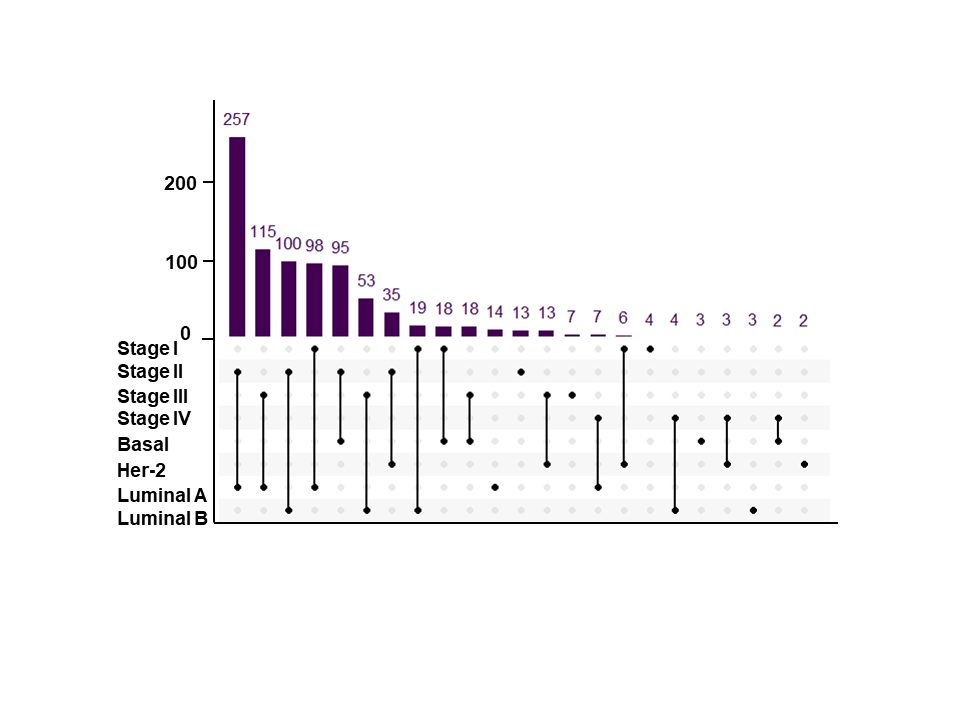

Supplement: Supplementary file 1 [file jcm-10-04071-s001.zip › jcm-1234077-supplementary/Supplementary Material Figure S5.PNG]

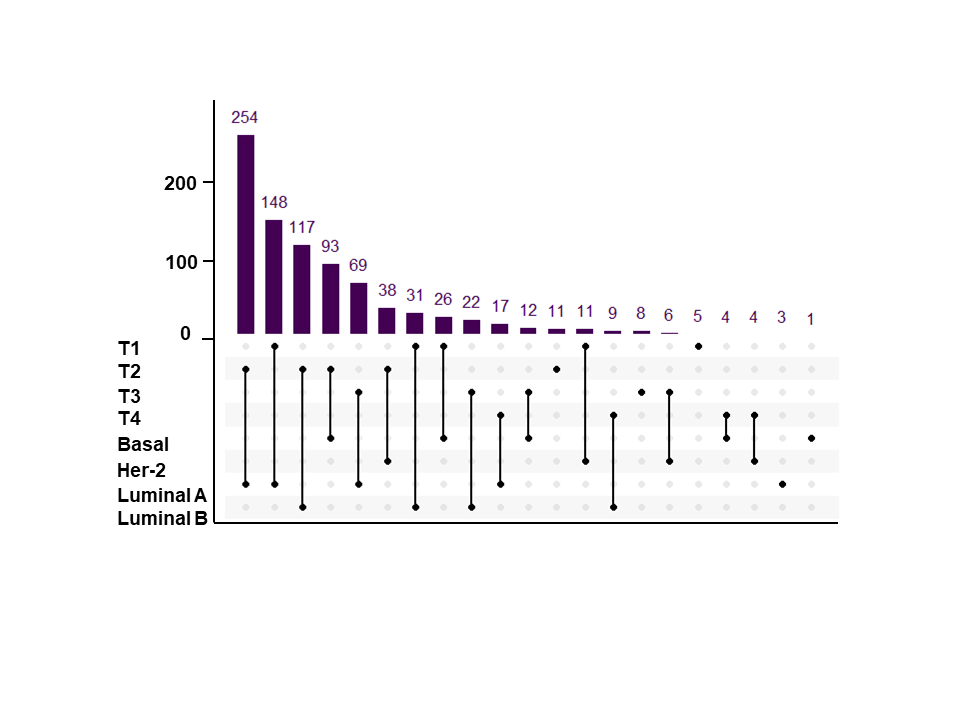

Supplement: Supplementary file 1 [file jcm-10-04071-s001.zip › jcm-1234077-supplementary/Supplementary Material Figure S6.PNG]

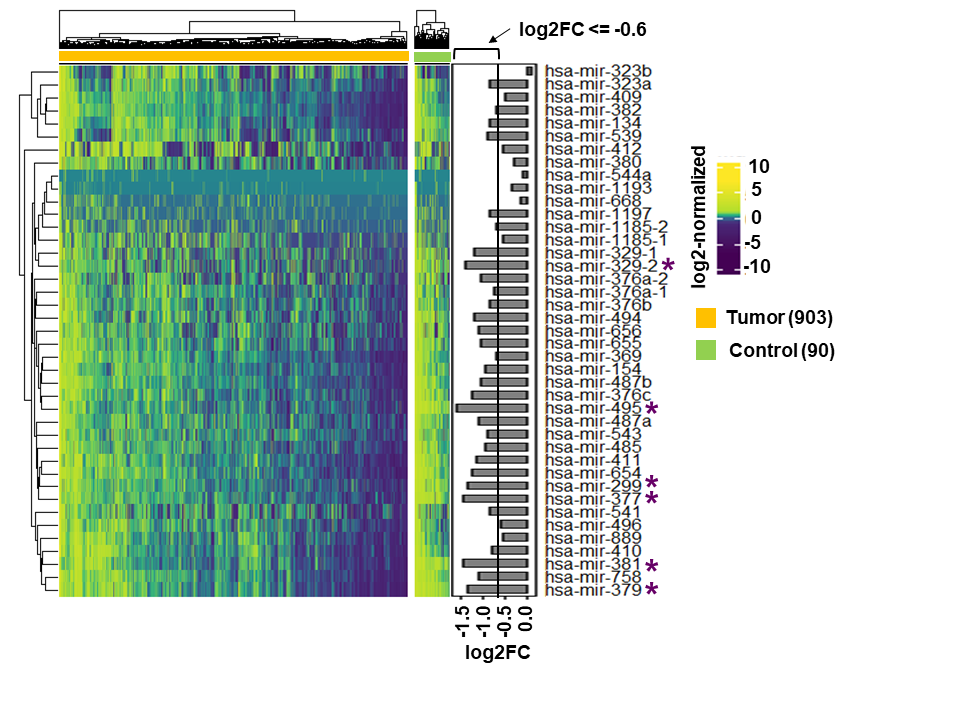

Supplement: Supplementary file 1 [file jcm-10-04071-s001.zip › jcm-1234077-supplementary/Supplementary Material Figure S7.PNG]

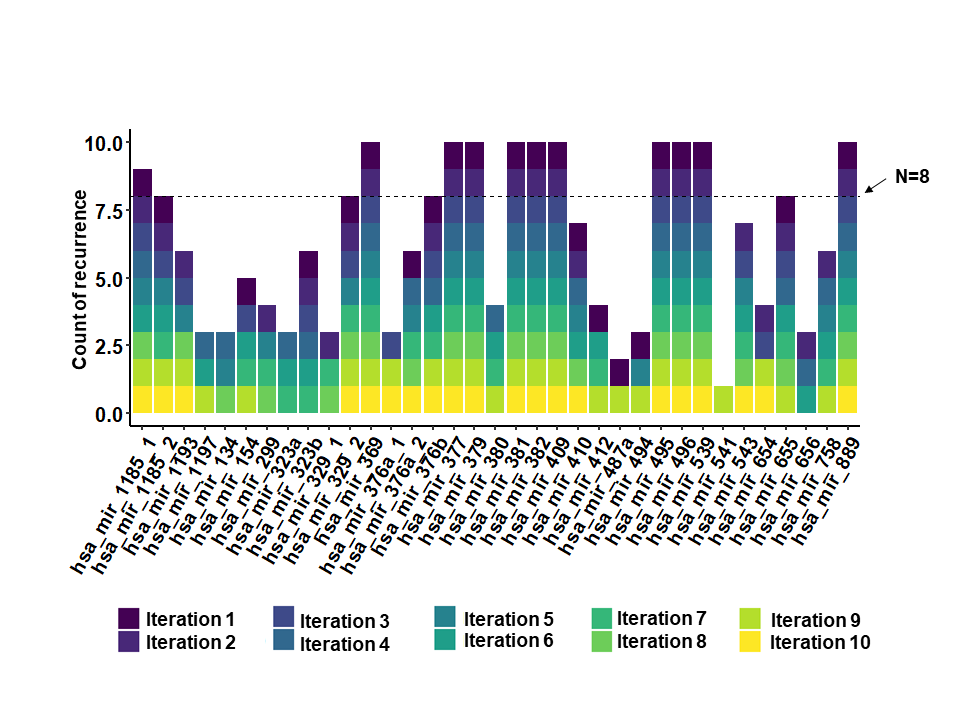

Supplement: Supplementary file 1 [file jcm-10-04071-s001.zip › jcm-1234077-supplementary/Supplementary Material Figure S8.PNG]
